# Age-Dependent Dysregulation of Muscle Vasculature and Blood Flow Recovery after Hindlimb Ischemia in the *mdx* Model of Duchenne Muscular Dystrophy

**DOI:** 10.3390/biomedicines9050481

**Published:** 2021-04-27

**Authors:** Paulina Podkalicka, Olga Mucha, Katarzyna Kaziród, Iwona Bronisz-Budzyńska, Sophie Ostrowska-Paton, Mateusz Tomczyk, Kalina Andrysiak, Jacek Stępniewski, Józef Dulak, Agnieszka Łoboda

**Affiliations:** Department of Medical Biotechnology, Faculty of Biochemistry, Biophysics and Biotechnology, Jagiellonian University, 30-387 Kraków, Poland; paulina.podkalicka@doctoral.uj.edu.pl (P.P.); olga.mucha@doctoral.uj.edu.pl (O.M.); katarzyna.kazirod@doctoral.uj.edu.pl (K.K.); iwona.bronisz-budzynska@doctoral.uj.edu.pl (I.B.-B.); sophie.ostrowska-paton@student.uj.edu.pl (S.O.-P.); mateusz.tomczyk@icgeb.org (M.T.); kalina.andrysiak@doctoral.uj.edu.pl (K.A.); jacek.stepniewski@uj.edu.pl (J.S.); jozef.dulak@uj.edu.pl (J.D.)

**Keywords:** DMD, Duchenne muscular dystrophy, angiogenesis, endothelial cells, hindlimb ischemia, retinal angiogenesis

## Abstract

Duchenne muscular dystrophy (DMD), caused by a lack of functional dystrophin, is characterized by progressive muscle degeneration. Interestingly, dystrophin is also expressed in endothelial cells (ECs), and insufficient angiogenesis has already been hypothesized to contribute to DMD pathology, however, its status in *mdx* mice, a model of DMD, is still not fully clear. Our study aimed to reveal angiogenesis-related alterations in skeletal muscles of *mdx* mice compared to wild-type (WT) counterparts. By investigating 6- and 12-week-old mice, we sought to verify if those changes are age-dependent. We utilized a broad spectrum of methods ranging from gene expression analysis, flow cytometry, and immunofluorescence imaging to determine the level of angiogenic markers and to assess muscle blood vessel abundance. Finally, we implemented the hindlimb ischemia (HLI) model, more biologically relevant in the context of functional studies evaluating angiogenesis/arteriogenesis processes. We demonstrated that both 6- and 12-week-old dystrophic mice exhibited dysregulation of several angiogenic factors, including decreased vascular endothelial growth factor A (VEGF) in different muscle types. Nonetheless, in younger, 6-week-old *mdx* animals, neither the abundance of CD31^+^α-SMA^+^ double-positive blood vessels nor basal blood flow and its restoration after HLI was affected. In 12-week-old *mdx* mice, although a higher number of CD31^+^α-SMA^+^ double-positive blood vessels and an increased percentage of skeletal muscle ECs were found, the abundance of pericytes was diminished, and blood flow was reduced. Moreover, impeded perfusion recovery after HLI associated with a blunted inflammatory and regenerative response was evident in 12-week-old dystrophic mice. Hence, our results reinforce the hypothesis of age-dependent angiogenic dysfunction in dystrophic mice. In conclusion, we suggest that older *mdx* mice constitute an appropriate model for preclinical studies evaluating the effectiveness of vascular-based therapies aimed at the restoration of functional angiogenesis to mitigate DMD severity.

## 1. Introduction

Duchenne muscular dystrophy (DMD), a genetic disease that predominantly affects boys already in early childhood, represents one of the most destructive types of muscular dystrophies. It is caused by a lack of functional dystrophin, as the result of more than 7000 patient-specific mutations in one of the largest human genes, *DMD*, containing 79 exons and approximately 2.4 million bp [1,2].

Dystrophin with its protein partners forms a large dystrophin–glycoprotein complex, abundantly expressed in the sarcolemma. Its absence is associated with higher susceptibility of muscles to damage by contractile forces, which eventually leads to their progressive degeneration [3]. Patients suffering from DMD live up to their 4th decade of life and die prematurely, mostly because of cardio–respiratory complications [4]. Despite the enormous effort and progress in the development of DMD treatment approaches aimed at the restoration of functional dystrophin, effective therapies that would be amenable for all patients still do not exist [5]. Hence, investigations of novel mediators, as well as an understanding of secondary consequences of dystrophin loss, are of great importance and may contribute to the invention of new therapeutic options ameliorating DMD pathology.

A growing body of evidence indicates that dystrophin, except for being an indispensable structural protein of muscles, acts as a mediator of intracellular signaling [6], and its expression appears not to be restricted to muscle cells. Accordingly, dystrophin presence has been demonstrated in muscle satellite cells (SCs) [7], fibroblasts [8], and also vascular smooth muscle cells [9], and endothelial cells (ECs) [10], playing a pivotal role in vascular homeostasis. Hence, alterations in tissue vascularization and the function of blood vessels under dystrophic conditions have already been proposed. Furthermore, dating back to the period before the discovery of dystrophin, grouped necrotic muscle fibers observed on histological specimens from DMD patients were believed to be the cause of insufficient delivery of oxygen and nutrients to muscles as the result of ischemia and impaired blood supply. Though confounding results obtained in this research area did not finally support the vascular hypothesis, some studies uncovered abnormalities in the blood vessel structure, including pale and swollen endothelial cells, an elevated area of capillaries with an occluded lumen, replication of the basement membrane around the vessels and the existence of degenerating and regenerating capillaries in the muscle biopsies of DMD patients, suggesting that vascular aberrations may, at least, contribute to the disease severity [11].

Since muscle biopsies from patients suffering from DMD and well-matched controls from healthy subjects are highly inaccessible, the vast majority of the more recent studies analyzed the status of angiogenesis predominantly in dystrophic animals [11]. A plethora of methods may be used to assess vascular abnormalities, ranging from evaluation of angiogenic factors in tissues or serum, determination of vasculature based on the immunofluorescent analyses to the most biologically relevant, functional studies. Among them, the hindlimb ischemia (HLI) model enables in vivo monitoring of blood flow restoration as the result of angiogenesis/arteriogenesis using the femoral artery ligation (FAL) procedure. Such an approach provides meaningful insight into functional angiogenesis with accompanying molecular events related to inflammation and regeneration occurring in the affected muscles [12,13]. Studies utilizing this method, however, revealed both increased [14] and decreased [15] angiogenic responses in the most commonly used mouse model of DMD, the *mdx* mice. Moreover, inconsistent results were demonstrated regarding muscle vascularization, which was shown to be either decreased [15] or almost normal [16] in animals lacking dystrophin, indicating still not fully understood and complicated alterations in vascular homeostasis.

Importantly, the discrepancies between several published studies might result, among others, from varying ages of the animals involved. Although the dystrophic phenotype of *mdx* mice does not fully recapitulate disease severity in DMD patients [17], key features of human myopathology are reported in such animals. Despite differences in the timeline of pathological changes reported by different groups, skeletal muscle degeneration appears at around 3 weeks of age, with the peak of muscle necrosis and robust regeneration by approximately 7 weeks of age. Afterward, muscle regeneration is considered to be rather of low intensity [18,19]. Massopust et al. revealed a progressive pattern of disease pathology related to myofibers damage and repair during the first year of life [20], appreciating the undermined relevance of *mdx* mice as a model of DMD pathology. In the context of angiogenic-related disturbances, though age-dependent effects were already suggested [11], a direct comparison of *mdx* mice of different ages in one study was not previously performed. The only comprehensive report investigating structural and functional aberrations of microvasculature in dystrophic mice of distinct ages was performed by Latroche et al., who analyzed *mdx*-4Cv mice (harboring nonsense, chemically-induced point mutation in exon 53 of *Dmd* gene) and revealed microvessel alterations mostly in 1-year-old mice [16].

Taking into account all the above together with the emerging, vascular-based therapies aimed at the attenuation of DMD symptoms [11,21], it becomes apparent that the clarification of an angiogenic status in *mdx* mice is still required. Considering this, we aimed to (i) verify if angiogenesis alterations truly exist in skeletal muscles of dystrophic mice focusing mostly on the functional assessment of blood flow restoration upon HLI and (ii) if those changes are in any way affected by the age of *mdx* animals. As we previously reported diminished expression of platelet endothelial cell adhesion molecule-1 (PECAM-1, CD31), a marker of ECs, as well as the level of vascular endothelial growth factor (VEGF) transcript [22] and protein [22,23,24] in skeletal muscles of 12-week-old *mdx* mice compared to wild-type (WT) animals, we hypothesized vascular abnormalities in skeletal muscles and impeded recovery from HLI of 12-week-old dystrophic mice, which was confirmed in the current work. Simultaneously, we demonstrated that despite the altered expression pattern of angiogenic genes in skeletal muscles of younger 6-week-old mice, the blood flow restoration was not compromised, supporting the hypothesis of age-dependent alterations in angiogenesis in dystrophic animals.

## 2. Materials and Method

### 2.1. Animal Models

All animal experiments were performed in accordance with national and European legislation, after approval by the 2nd Institutional Animal Care and Use Committee (IACUC) in Kraków, Poland (approval numbers: 276/2017 (12 October 2017), 231/2018, 232/2018 (19 July 2018)). Animals were housed in specific pathogen-free (SPF) conditions with water and food available ad libitum. *mdx* mice C57BL/10ScSn-*Dmd^mdx^*/J and wild-type (WT) mice C57BL/10ScSnJ were purchased from the Jackson Laboratory (stocks no. 001,801 and 000476, respectively). Mice were bred on a mixed C57BL/10ScSn and C57BL/6×FVB background, as we described previously [25]. Genotyping of animals was performed by PCR on the DNA isolated from the tail tips (A&A Biotechnology, Gdańsk, Poland) according to the established method [26]. 6- and 12-week-old dystrophic and age-matched WT male mice were used for experiments.

### 2.2. Blood Cell Count

Blood was collected from the vena cava directly to ethylenediaminetetraacetic acid (EDTA)-coated tubes and was analyzed by the scil Vet abc (HORIBA, Kyoto, Japan). The white blood cells (WBC) number was determined in 12-week-old WT and *mdx* mice: untreated and 7 and 21 days after HLI. Additionally, the percentage of granulocytes, monocytes, and lymphocytes among WBC was calculated in 12-week-old WT and *mdx* mice 7 and 21 days after HLI.

### 2.3. In Vitro Angiogenesis Assay

The influence of WT and *mdx*-derived serum on ECs tube formation was assessed by endothelial tube formation assay. Per each well of a 96-well plate, 80 μL of Matrigel^®^ Matrix Basement Membrane (Growth Factor Reduced, Corning, Corning, NY, USA) was added and incubated at 37 °C for 30 min. One thousand human aortic endothelial cells (HAECs, Gibco, Dublin, Ireland) were seeded on polymerized Matrigel in 100 μL of Endothelial Cell Basal Medium MV 2(PromoCell, Heidelberg, Germany) enriched with 10% serum from WT and *mdx* mice (technical duplicates for each mouse were prepared). Tube formation was investigated after 5 h by phase-contrast microscopy (Nikon Eclipse Ti, Nikon, Japan). ImageJ with an Angiogenesis Analyzer was used to quantify the number of nodes, junctions, and branches formed by HAECs [27].

### 2.4. Forelimb Grip Strength Assessment

The measurement of the forelimb grip strength was performed in 12-week-old animals according to our previous study [23].

### 2.5. HLI Induction

Unilateral left femoral artery ligation (FAL) was carried out as described before [28,29] with modifications. Mice were anesthetized by intraperitoneal injection of 100 mg/mL ketamine (Bioketan, Vetoquinol, Poland), 20 mg/mL xylazine (Biowet, Puławy, Poland), and saline in a ratio of 1:0.5:8.5 (10 μL per gram of body weight (BW)). Fur was removed from the hindlimbs using hair removal cream, and the skin was sanitized with betadine (Prolab, Paterek, Poland) and 70% alcohol swabs. Mice were warmed for 5 min, and blood flow just before FAL was measured in both hindlimbs using laser speckle contrast imaging (PeriCam PSI System, Perimed, Järfälla, Sweden). Next, the mice were placed on a heated (37 °C) operation table, and a small incision was made in the middle of the left thigh. Fatty tissue was then pulled aside until the neurovascular bundle was visible. The femoral artery was gently separated from the femoral vein and nerve and was subsequently occluded with a 6–0 silk suture in two distinct regions. The incision was closed by surgical sutures followed by the application of betadine. After the procedure, the mice were warmed for 5 min, and the blood perfusion from ischemic and uninjured, non-ischemic (control) limbs were measured (day 0). An analgesic (0.08 mg/kg BW buprenorphine) was administered subcutaneously right after FAL twice daily for two consecutive days. Blood flow measurements were repeated 3, 7, and 21 days after FAL as described above. The mice were sacrificed 7 and 21 days after the procedure, and the biological samples (muscles, blood) were collected for downstream analyses. No necrotic toes were observed in all animals subjected to FAL throughout the entire experiment. The results were calculated at each time-point as the ratio of ischemic to non-ischemic foot blood perfusion and normalized to the measurements before FAL. Additionally, blood flow recovery rate 7 and 21 days after HLI was presented in relation to measurement right after HLI induction. Basal blood perfusion was presented as the average value of blood flow of two limbs as measured right before FAL.

### 2.6. Generation and Differentiation of Human-Induced Pluripotent Stem Cells (hiPSCs)-Derived Skeletal Muscle Cells

All hiPSCs lines in this study were generated with the use of Sendai vectors (CytoTune-iPS Sendai Reprogramming Kit, ThermoFisher Scientific, Waltham, MA, USA). Control hiPSCs (HPSI1013ikuxp_1 line) from male skin fibroblasts were obtained from The Human Induced Pluripotent Stem Cells Initiative’s collection (www.hipsci.org, accessed on 9 January 2017) or were generated from peripheral blood mononuclear cells (PBMCs) isolated from a healthy volunteer as described by Stępniewski et al. [30]. The DMD hiPSCs line was reprogrammed from fibroblasts derived from a DMD patient carrying a mutation in gene encoding dystrophin (deletion of DMD exon 45) purchased from the Coriell Institute for Medical Research. Subsequently, all hiPSCs lines were characterized to confirm their pluripotency and differentiated to skeletal muscle cells using the Genea Biocells Skeletal Muscle Differentiation Kit according to the manufacturer’s protocol, as described by us previously [25]. Briefly, 10,000 cells were seeded onto collagen I-coated wells of a 24-well plate and cultured for 10 days in a Skeletal Muscle Induction Medium, refreshed every 2–3 days. Subsequently, the cells were reseeded at a density of 5000 cells per cm^2^ onto collagen I-coated wells and maintained in a Skeletal Myoblast Medium for the next 7–8 days with media change every other day. After this time, the medium was switched to a Myotube Medium and changed every other day for the next 6 days. After day 18 of differentiation, the cells were collected, and RNA was isolated.

### 2.7. RNA Isolation, Reverse Transcription (RT), and Quantitative Real-Time PCR (qRT-PCR)

The Chomczynski-Sacchi method [31] was used to isolate RNA, as in our previous study [25]. Muscles were collected in tubes containing RNAlater (Sigma–Aldrich, St. Louis, MO, USA), immediately snap-frozen in liquid nitrogen, and stored at −80 °C for downstream analyses. The concentration and quality of RNA were determined with NanoDrop 1000 Spectrophotometer (ThermoFisher Scientific, Waltham, MA, USA) followed by the RT reaction using recombinant M-MuLV reverse transcriptase (ThermoFisher Scientific, Waltham, MA, USA). qRT-PCR was performed as described previously [25] using StepOne Plus Real-Time PCR (Applied Biosystems, ThermoFisher Scientific, Waltham, MA, USA) with cDNA, SYBR Green PCR Master Mix (Sigma–Aldrich, St. Louis, MO, USA), and specific primers (listed in Table 1). Eukaryotic elongation factor 2 (*Eef2*) was used as a housekeeping gene, and no differences in *Eef2* level between the analyzed groups were observed. Relative quantification of gene expression was calculated based on the comparative threshold cycle value (Ct) method using the equation 2^−ΔCt^, where ΔCt = Ct_gene of interest_ − Ct*_Eef2_*. The results were presented as a fold change in comparison to WT animals in case of non-HLI conditions or as a fold change in comparison to non-ischemic limb after HLI.

### 2.8. Histological and Immunofluorescent Analysis of Muscles

For a histological assessment of inflammation (hematoxylin and eosin, H&E) and centrally nucleated fibers (CNF) abundance, muscles were collected and pre-treated with an optimal cutting temperature (OCT) medium (Leica, Wetzlar, Germany) for a few minutes directly after collection. Afterward, they were transferred to new, OCT-containing tubes, frozen in liquid nitrogen-cooled isopentane, and stored at −80 °C. Then, 8–10 µm thick sections were cut with a cryotome (Leica, Wetzlar, Germany), air-dried for at least 2 h, and kept at −20 °C for further analyses. H&E staining was performed and analyzed according to our previous studies [24,25,32] after taking pictures of the whole tissues. The assessment of the inflammation extent was conducted using arbitrary units, according to our previous expertise [25]. An analysis of the CNF percentage indicating the level of regeneration was performed based on H&E staining—pictures of the whole muscle’s tissue section were taken, and the percentage of CNF among all fibers was calculated.

Immunofluorescent staining of CD31 and alpha-smooth muscle actin (α-SMA) was performed as previously described with slight modifications [29]. Primary antibodies: rat anti-mouse CD31 (BD Pharmingen, San Diego, CA, USA, 550274, 1:200) and rabbit anti-human α-SMA (Abcam, Cambridge, UK, ab5694, 1:200) were used, followed by incubation with secondary antibodies: goat anti-rat Alexa Fluor 488 for detection of CD31 in green and goat anti-rabbit Alexa Fluor 568 for detection of α-SMA in red (both from ThermoFisher Scientific, Waltham, MA, USA, 1:500). Negative controls were prepared by omitting primary antibody addition. Pictures of the whole tissue were taken, and CD31^+^α-SMA^+^ double-positive vessels were analyzed quantitatively per muscle area.

The stainings were visualized under a Nikon Eclipse Ti or Leica DMI 6000B fluorescent microscope. All histological assessments were analyzed by the investigator blind to the mice genotype using ImageJ or LAS X software. If needed, the brightness and/or contrast were adjusted for all of the pictures equally.

### 2.9. Determination of Serum Creatine Kinase (CK) and Lactate Dehydrogenase (LDH) Activity

The activity of CK and LDH was measured using diagnostic Liquick Cor-CK and Liquick Cor-LDH kits (Cormay, Warszawa, Poland), respectively, according to the vendor’s instruction. Blood was collected from the vena cava. Clear, non-hemolysed serum was prepared by allowing the blood to clot at room temperature for 30 min and then centrifuged at 2000× *g* for 10 min at 4 °C. Serum samples were diluted 10 times for the assay. The absorbance values were then converted to LDH or CK activity (U/l) using the formula supplied with the kit and presented as a fold change in relation to WT animals.

### 2.10. Enzyme-Linked Immunosorbent Assay (ELISA)

Protein lysates isolation from snap-frozen in liquid nitrogen muscles was performed by homogenization of tissues in 1% TritonX100 in PBS using TissueLyser (QIAGEN, Hilden, Germany). After centrifugation (7000× *g*, 10 min, 4 °C), the supernatant was collected, and the total protein concentration was determined by bicinchoninic acid (BCA, Sigma–Aldrich, St. Louis, MO, USA) assay. VEGF, CD105 (endoglin), and stromal cell-derived factor-1 (SDF-1) levels in muscle tissues were determined according to the vendor’s instructions (R&D Systems, Minneapolis, MN, Canada) using 100 µg of protein lysate. The osteopontin (OPN) level in serum was quantified according to the manufacturer’s protocol (R&D Systems, Minneapolis, MN, Canada), with the serum diluted 750 times for the assay.

### 2.11. Flow Cytometry Analysis

The preparation of cells isolated from hindlimb muscles from both legs for flow cytometry analysis was performed as described previously [24,25,32]. For the examination of fibro-adipogenic progenitors (FAPs; CD45^−^CD31^−^Sca-1^+^CD34^+^ cells), pericytes (CD45^−^CD31^−^CD34^−^CD146^+^ cells), and different subpopulations of ECs (CD45^−^CD31^+^Sca-1^+^, CD45^−^CD31^+^CD146^+^, CD45^−^CD31^+^CD34^+^ and CD45^−^CD31^+^CD34^+^Sca-1^+^ cells) the following antibodies were used: rat anti-mouse CD34-PE (1:30, clone RAM34), rat anti-mouse CD45-BV605 (1:30, clone 30-F11), rat anti-mouse CD31-BV786 (1:30, clone 390), rat anti-mouse Sca1-APC-Cy7 (1:30, clone D7) (all from BD Biosciences, San Jose, CA, USA), and rat anti-mouse CD146-PE-Cy7 (1:30, clone ME-9F1) (BioLegend San Diego, CA, USA). Cells were additionally stained with 10 μg/mL Hoechst 33,342 (Sigma–Aldrich, St. Louis, MO, USA). The analysis was performed using an LSRFortessa flow cytometer with FACSDiva (BD Biosciences, San Jose, CA, USA) based on the appropriate fluorescence minus one (FMO) controls.

### 2.12. Statistical Analyses

Data are presented as mean ± SEM. Differences between groups were tested for statistical significance using the unpaired 2-tailed Student’s *t*-test for comparing of two groups or the one-way or two-way ANOVA followed by Tukey’s posthoc test when more than two groups were analyzed; *p* < 0.05 was considered significant and *p* = 0.05–0.1 as a tendency. The outliers were identified based on Grubb’s test, and the *n* number indicating the number of animals per group was included in the figure legend.

## 3. Results

### 3.1. Alterations in Angiogenesis Markers Are Present in Skeletal Muscles of 6- and 12-Week-Old Dystrophic Mice

*Mdx* mouse with the spontaneous, nonsense, point mutation in exon 23 of the dystrophin gene is the most commonly studied animal model of DMD pathogenesis [33]. Although the *mdx* phenotype is considered mild compared to humans [34], several features of the disease are well recapitulated in those animals, as also reported in our recent studies [22,23,24,25]. In the current work, dystrophic mice exhibited decreased muscle functionality in a forelimb grip strength test (Figure S1a), which was accompanied by increased body weight (Figure S1b) and an abundance of CD45^−^CD31^−^Sca-1^+^CD34^+^ FAPs evaluated by flow cytometry in the gastrocnemius muscle (Figures S1c and S2).

**Figure 1:**
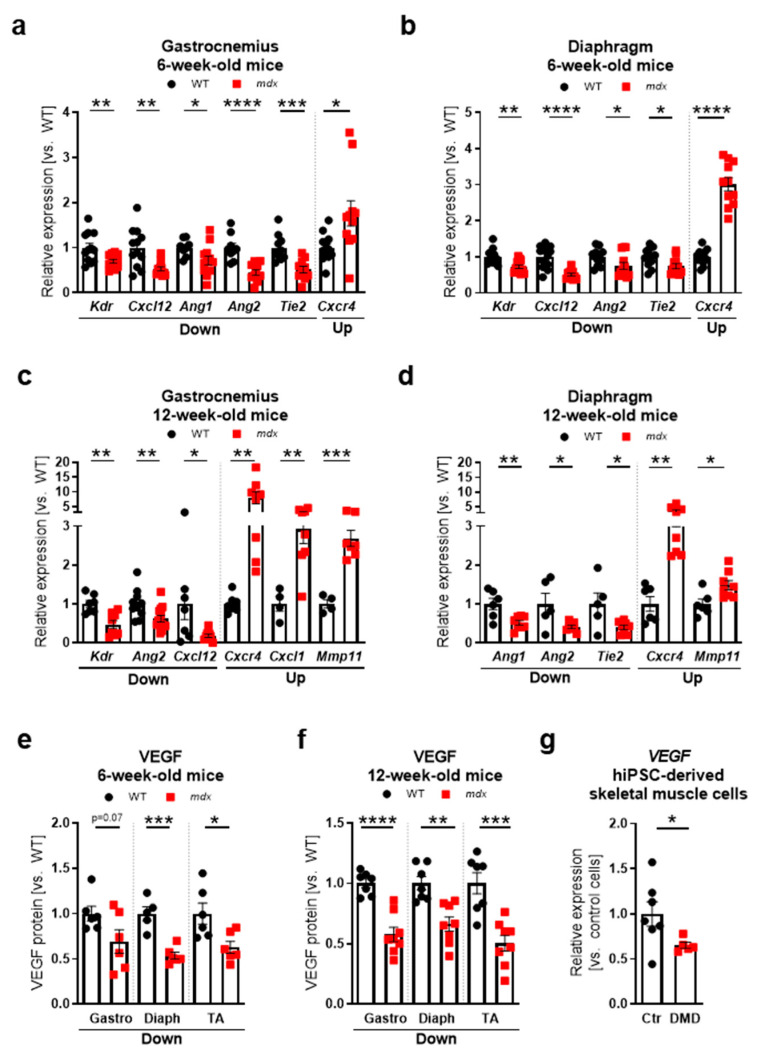

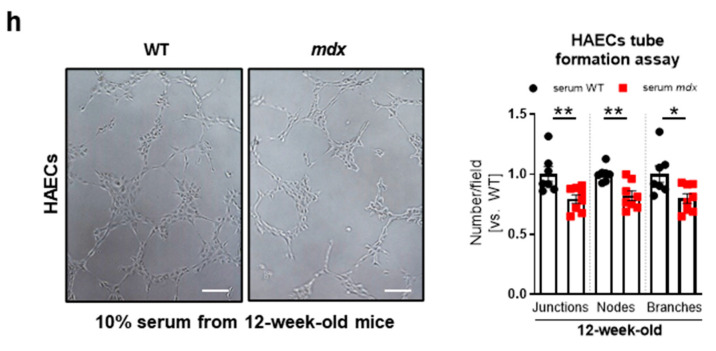
Alterations in angiogenesis markers in Duchenne muscular dystrophy (DMD) models. (**a**) The expression of *Kdr*, *Cxcl12*, *Ang1*, *Ang2*, and *Tie2* is decreased, whereas *Cxcr4* is increased in the gastrocnemius of 6-week-old *mdx* mice; qRT-PCR; *n* = 8–12/group. (**b**) In the diaphragm of 6-week-old dystrophic mice, the mRNA transcripts of *Kdr*, *Cxcl12*, *Ang2*, and *Tie2* are downregulated, whereas *Cxcr4* is upregulated, qRT-PCR; *n* = 11–12/group. (**c**) The expression of *Kdr*, *Ang2*, *Cxcl12* is decreased, whereas *Cxcr4*, *Cxcl1*, *Mmp11* is increased in the gastrocnemius of 12-week-old *mdx* mice; qRT-PCR; *n* = 4–13/group. (**d**) In the diaphragm of 12-week-old dystrophic mice, the mRNA transcripts of *Ang1*, *Ang2*, and *Tie2* are lower, whereas *Cxcr4* and *Mmp11* are upregulated; qRT-PCR; *n* = 5–8/group. The downregulated level of vascular endothelial growth factor (VEGF) protein is found in the dystrophic gastrocnemius (Gastro), diaphragm (Diaph), and tibialis anterior (TA) muscle of (**e**) 6-week-old and (**f**) 12-week-old animals; enzyme-linked immunosorbent assay (ELISA); *n* = 5–8/group. (**g**) Decreased expression of *VEGF* is present in the dystrophic (DMD) human induced pluripotent stem cells (hiPSC)-derived skeletal muscle cells in comparison to control (Ctr) cells; qRT-PCR; *n* = 5–7/group. (**h**) Human aortic endothelial cells (HAECs) tube formation is decreased in the presence of a medium containing 10% serum from 12-week-old *mdx* mice as presented in representative photos of tubular structures on Matrigel with quantification of nodes, junctions, and branches, formed by the cells after 5 h of incubation either with wild-type (WT) or *mdx*-mice derived serum; a microscopic assessment using a Nikon Eclipse microscope followed by ImageJ quantification using an Angiogenesis Analyzer. Scale bar: 100 μm; *n* = 7–8/group. The results are presented as mean ± SEM. * *p* < 0.05, ** *p* < 0.01, *** *p* < 0.005, **** *p* < 0.0001 with unpaired two-tailed Student’s *t*-test.

Furthermore, an elevated level of lactate dehydrogenase (LDH, Figure S1d) and creatine kinase (CK, Figure 1e) activity in serum, typical for dystrophic pathology [22,23,24,25,35], was observed. Additionally, an increased level of osteopontin (OPN), a recently described biomarker of DMD [36,37,38], was detected in the serum (Figure S1f) and in the gastrocnemius, where its transcript (*Spp1*) was upregulated almost 150-fold in comparison to WT mice (Figure S1g). OPN has been reported to contribute to multiple processes, including inflammation, fibrosis, and also angiogenesis [39]. Our examination revealed dysregulated mRNA levels of different angiogenic-related genes in the gastrocnemius (Figure S1a,c) and diaphragm (Figure 1b,d) of 6- and 12-week old dystrophic mice without a clear age- or muscle type-dependent pattern. Importantly, similar to our previous reports [22,23,24], the protein level of a major angiogenic factor, VEGF, was markedly decreased in the gastrocnemius, diaphragm, and tibialis anterior of both 6- and 12-week-old *mdx* mice (Figure 1e,f, respectively), indicating a complex regulation of different angiogenic pathways in skeletal muscles of dystrophic animals. Importantly, diminished expression of *VEGF* was also observed in dystrophic skeletal muscle cells differentiated from human induced pluripotent stem cells (hiPSCs) (Figure 1g).

Finally, we explored the angiogenic potential of serum obtained from 12-week-old WT and *mdx* animals using in vitro endothelial tube formation assay. It appeared that HAECs arranged well-formed, vessel-like tubular structures when cultured in a medium enriched in 10% serum from WT mice. However, supplementation with the serum of *mdx* mice decreased tube formation, as shown by the representative photos and the quantification of the number of nodes, junctions, and branches (Figure 1h).

### 3.2. Increased Number of Blood Vessels Is Predominantly Observed in Skeletal Muscles of 12-Week-Old, but Not 6-Week-Old Dystrophic Animals

**Figure 2:**
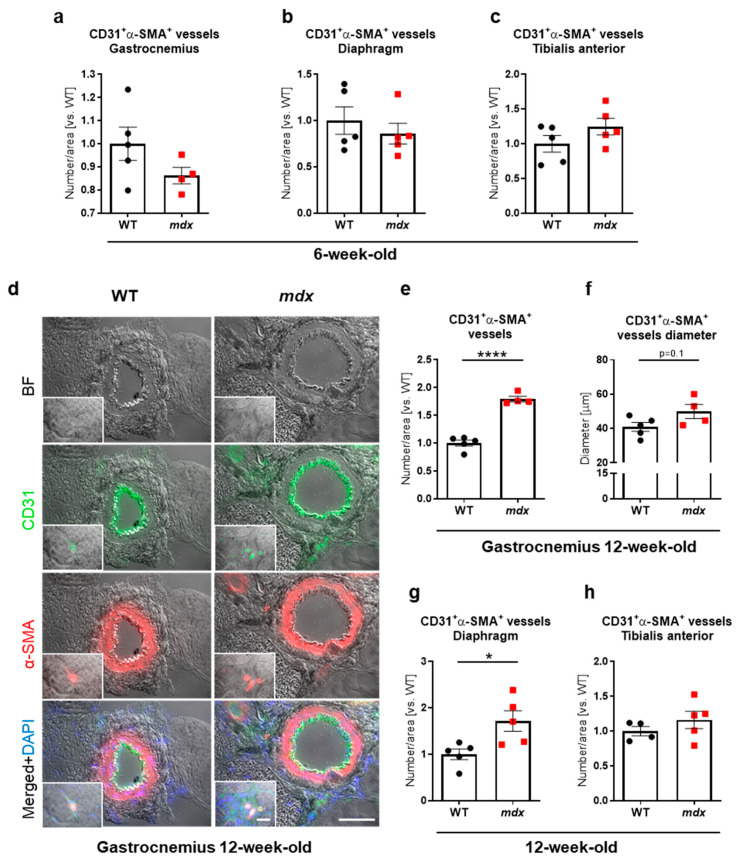
Blood vessel abundance in skeletal muscles of 6- and 12-week-old WT and *mdx* mice. In 6-week-old animals, the abundance of CD31+α-SMA+ double-positive vessels was not changed in the dystrophic (**a**) gastrocnemius, (**b**) diaphragm, and (**c**) tibialis anterior; quantitative analysis based on immunofluorescent staining; *n* = 4–5/group. (**d**) Representative pictures from immunofluorescent staining of CD31^+^α-SMA^+^ double-positive blood vessels in the gastrocnemius muscle of 12-week-old mice together with quantification indicating (**e**) elevated number of CD31^+^α-SMA^+^ double-positive blood vessels and (**f**) a tendency for increased blood vessel diameter in dystrophic mice. The number of CD31^+^α-SMA^+^ double-positive vessels assessed in (**g**) the diaphragm and (**h**) tibialis anterior of 12-week-old animals. Scale bar: 10 µm; bright field (BF). The results are presented as mean ± SEM. * *p* < 0.05, **** *p* < 0.0001 with unpaired two-tailed Student’s *t*-test.

In the next step, we sought to precisely determine the blood vessel abundance in different muscle types of 6- and 12-week-old WT and *mdx* mice. CD31 is expressed not only by ECs building blood vessels but also by immune cells [40,41] robustly infiltrating dystrophic muscles. Hence, the analysis of cells positive only for CD31 might lead to overinterpretation of obtained data. Being aware of this, we decided to use CD31 together with α-SMA as blood vessel markers. A detailed analysis of immunofluorescent staining of CD31^+^α-SMA^+^ double-positive blood vessels in the gastrocnemius (Figure 2a), diaphragm (Figure 2b), and tibialis anterior (Figure 2c) did not reveal any changes between 6-week-old WT and *mdx* mice.

Interestingly, older animals demonstrated an elevated number of CD31^+^α-SMA^+^ double-positive blood vessels (Figure 2d,e) with a slightly increased vessel diameter (Figure 2f) in the gastrocnemius of 12-week-old *mdx* mice. Of note, the accumulation of CD31^+^α-SMA^+^ double-positive vessels was predominantly observed in the cell infiltration/muscle degeneration areas (Figure 2d, small pictures). Similarly, the vessel number was also increased in the diaphragm (Figure 2g), but not in the tibialis anterior (Figure 2h), indicating muscle-type specific changes in the abundance of blood vessels in 12-week-old mice.

**Figure 3:**
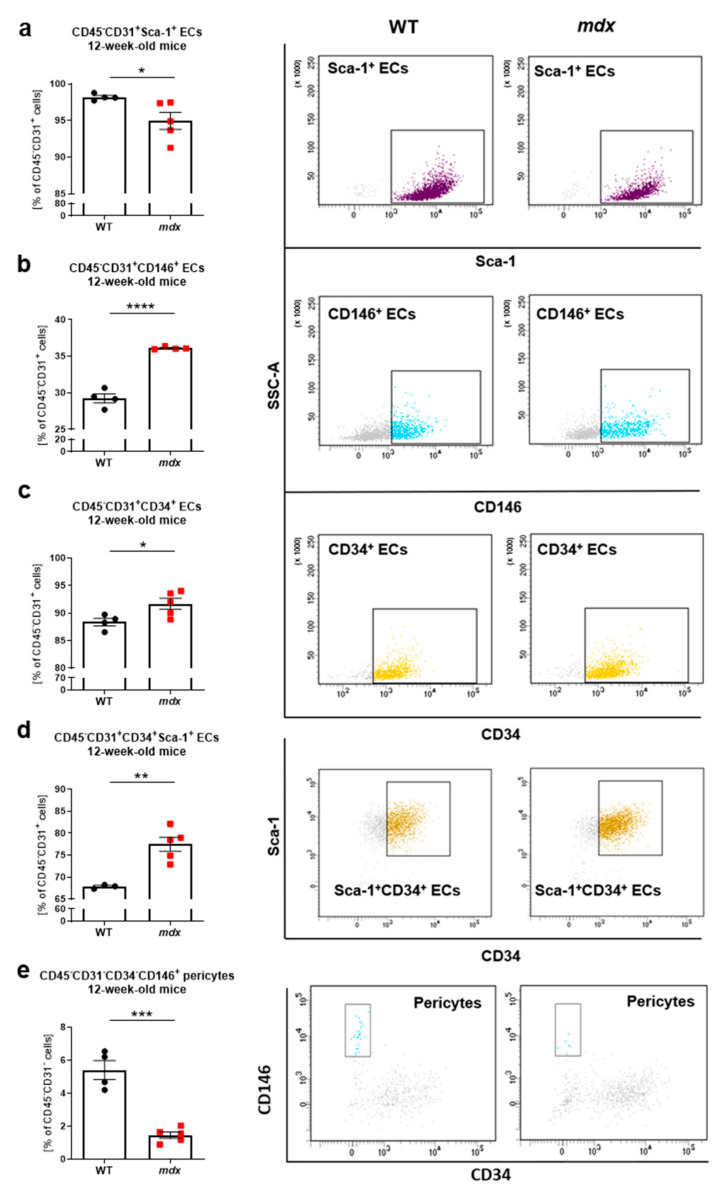
Flow cytometry-based analysis of endothelial cells (ECs) and pericytes in hindlimb muscles of 12-week-old WT and *mdx* animals. The analysis of subtypes of ECs: (**a**) CD45^−^CD31^+^Sca-1^+^, (**b**) CD45^−^CD31^+^CD146^+^, (**c**) CD45^−^CD31^+^CD34^+^, and (**d**) CD45^−^CD31^+^CD34^+^Sca-1^+^ of WT and *mdx* mice. Flow cytometry analysis calculated as a percentage of CD45^−^CD31^+^cells and representative two-parameters flow cytometry dot plots; *n* = 4–5/group. (**e**) The number of pericytes identified as CD45^−^CD31^−^CD34^−^CD146^+^ cells; flow cytometry analysis calculated as the percentage of CD45^−^CD31^−^ cells and representative two-parameters flow cytometry dot plots; *n* = 4–5/group. The results are presented as mean ± SEM. * *p* < 0.05, ** *p* < 0.01, *** *p* < 0.005, **** *p* < 0.0001 with unpaired two-tailed Student’s *t*-test.

When we further phenotyped CD45^−^CD31^+^ ECs from hindlimb muscles of 12-week-old mice using flow cytometry (Figure S2), it appeared that the majority of ECs possessed a Sca-1 marker (Figure 3a) with a subtly declined percentage in *mdx* ECs, whereas CD45^−^CD31^+^CD146^+^ (Figure 3b), CD45^−^CD31^+^CD34^+^ (Figure 3c), and CD45^−^CD31^+^CD34^+^Sca-1^+^ (Figure 3d) ECs subpopulations were slightly increased in *mdx* vs. WT animals. Simultaneously, a diminished level of CD45^−^CD31^−^CD34^−^CD146^+^ pericytes was demonstrated in *mdx* animals (Figure 3e), suggesting possible alterations in the functionality of blood vessels.

### 3.3. Basal Blood Perfusion and Revascularisation in Response to HLI Are Decreased in 12-Week-Old, but Not 6-Week-Old mdx Mice

**Figure 4:**
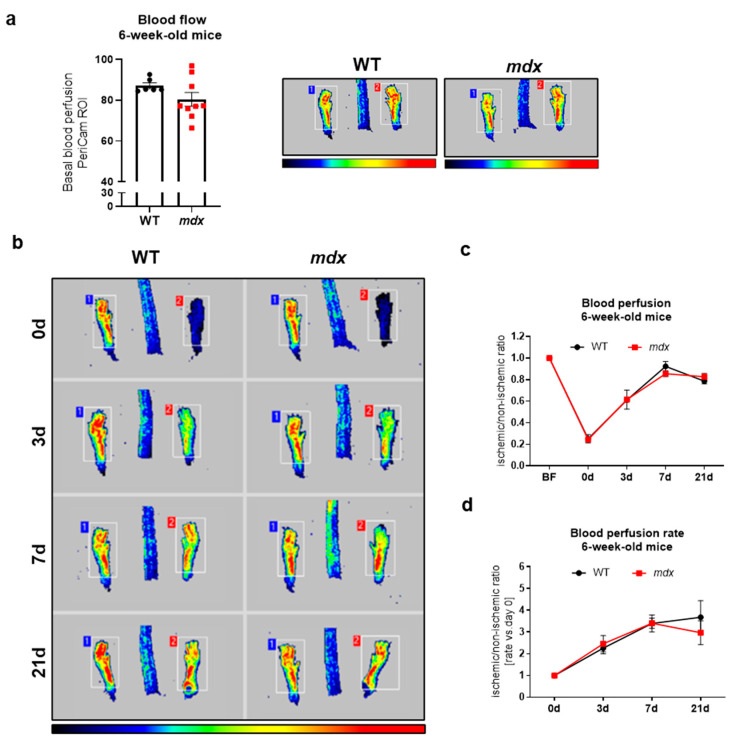
Basal blood flow and revascularization in 6-week-old WT and *mdx* animals following hindlimb ischemia (HLI). (**a**) Hindlimb blood flow of 6-week-old wild-type and dystrophic animals; PeriCam measurement; region of interest (ROI); *n* = 6–9/group. (**b**) The recovery of blood flow in WT and *mdx* mice in the following days after the HLI procedure shown by representative pictures and (**c**) calculation of blood perfusion and (**d**) blood perfusion recovery rate; *n* = 5–10/group. The results are presented as mean ± SEM. The statistical significance was tested with (**a**) unpaired two-tailed Student’s *t*-test or (**c**,**d**) two-way ANOVA with Tukey’s posthoc test.

To evaluate possible functional abnormalities, we utilized the complex HLI model of angiogenesis and arteriogenesis. In general, ligation of the unilateral left femoral artery causes a drastic reduction in blood flow, and its restoration might be monitored at several time points after HLI. In our analyses, we measured blood perfusion recovery at 3, 7, and 21 days following FAL, and we presented the results either as values of an ischemic/non-ischemic limb ratio or as a rate of blood perfusion increase vs. day 0 to better visualize the magnitude of observed changes. The measurement of basal blood perfusion performed in 6-week-old mice before the induction of HLI (Figure 4a) as well as its recovery after FAL (Figure 4b–d) did not reveal any changes, despite prominent variations in the level of numerous angiogenic-related genes in the gastrocnemius (Figure 1a) and diaphragm (Figure 1b).

**Figure 5:**
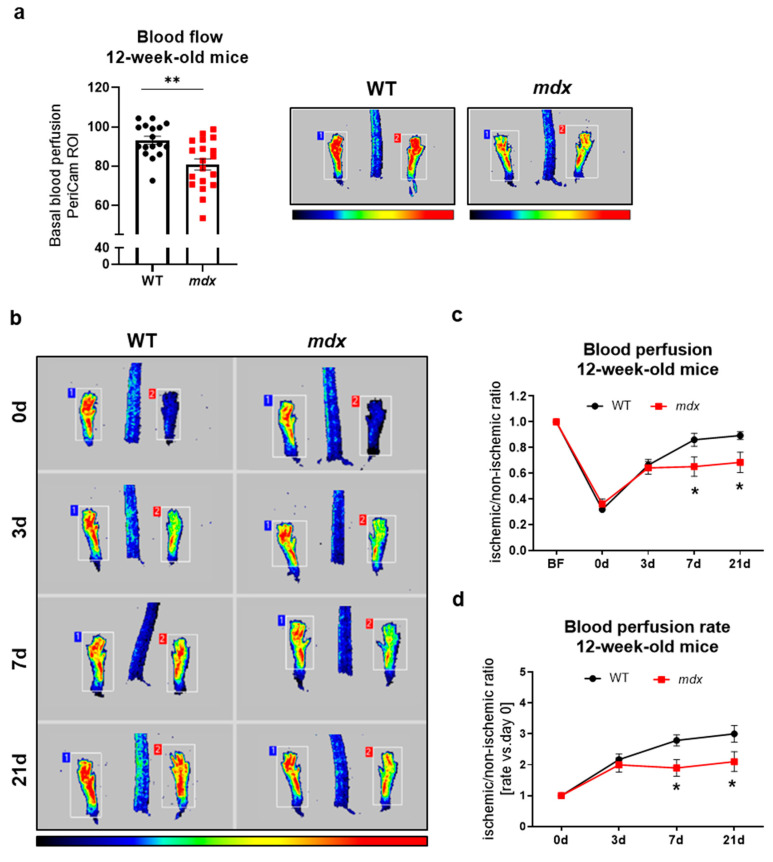
Decreased revascularization in 12-week-old *mdx* mice following hindlimb ischemia (HLI). (**a**) Under basal conditions, hindlimb blood flow is lower in dystrophic animals; PeriCam measurement; region of interest (ROI); *n* = 17–19/group. (**b**) The recovery of blood flow is impaired in *mdx* mice both 7 days and 21 days after the HLI procedure as shown by representative pictures and a calculation of (**c**) blood perfusion and (**d**) blood perfusion rate; *n* = 11–14/group. The results are presented as mean ± SEM. * *p* < 0.05, ** *p* < 0.01 with (**a**) unpaired two-tailed Student’s *t*-test or (**c**–**d**) with two-way ANOVA with Tukey’s posthoc test.

An analysis of 12-week-old animals demonstrated significantly lower basal blood perfusion in *mdx* animals (Figure 5a). After HLI induction, no changes in mice of both genotypes were noticed on day 3 after surgery, but a disturbed blood perfusion recovery was found in dystrophic animals starting from day 7 (Figure 5b–d). A significant difference was still evident on day 21 after HLI induction, indicating impaired blood flow restoration in *mdx* animals (Figure 5c,d). The above results may indicate an age-dependent decline in functional angiogenesis as a result of dystrophin deficiency.

**Figure 6:**
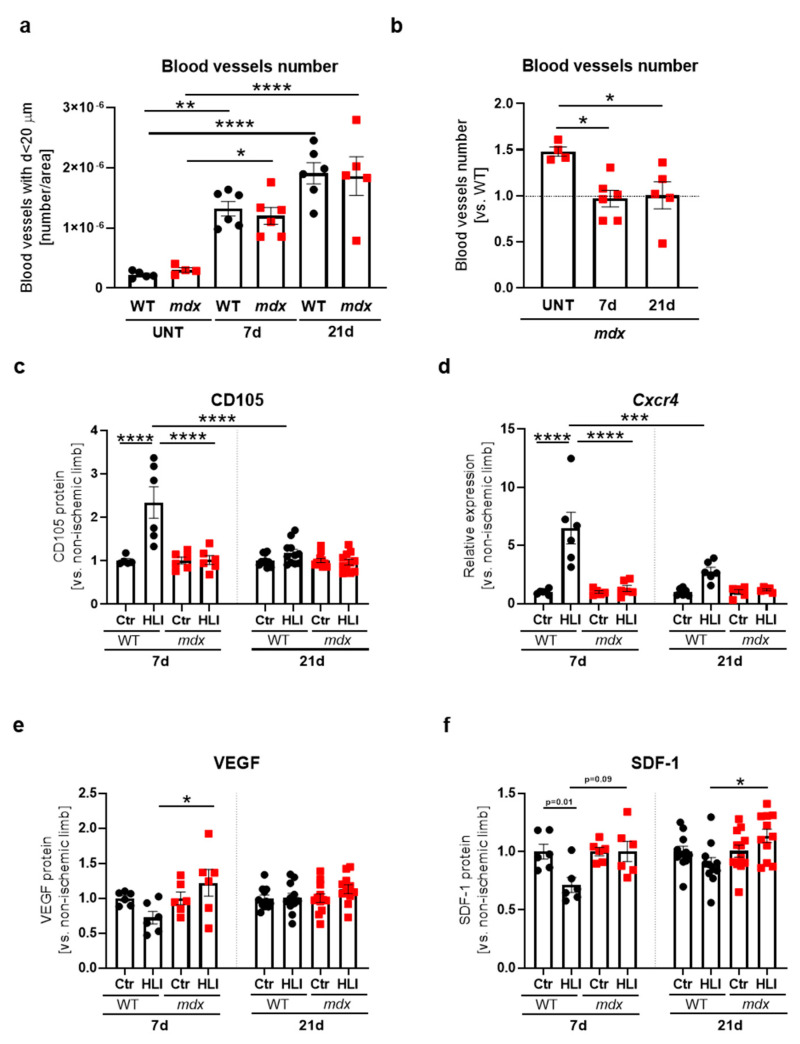
Blood vessels and angiogenic markers in the gastrocnemius of 12-week-old WT and dystrophic mice following hindlimb ischemia (HLI). (**a**) The number of blood vessels with a diameter lower than 20 µm in untreated (UNT) WT and *mdx* mice as well as in animals 7 and 21 days after HLI. (**b**) The total number of blood vessels in *mdx* mice shown in relation to WT mice after HLI; quantification based on immunofluorescent staining of CD31^+^α-SMA^+^ double-positive blood vessels; *n* = 4–6/group. (**c**) The protein level of CD105; ELISA, *n* = 5–11/group and (**d**) *Cxcr4* transcript; qRT-PCR, *n* = 5–6/group. (**e**) Vascular endothelial growth factor (VEGF); ELISA, *n* = 6–12/group and (**f**) stromal derived factor-1 (SDF-1); ELISA; *n* = 6–12/group in the gastrocnemius of WT and *mdx* mice 7 and 21 days after HLI. The results are presented as mean ± SEM. * *p* < 0.05, ** *p* < 0.01, *** *p* < 0.005, **** *p* < 0.0001 by one-way ANOVA with Tukey’s posthoc test.

In the next steps, we wanted to investigate the impaired blood flow recovery of 12-week-old dystrophic animals deeper by analyzing markers of ischemia-induced angiogenesis/arteriogenesis, inflammation, regeneration, and fibrosis following FAL. A prominent increase in the number of CD31^+^α-SMA^+^ double-positive blood vessels with a diameter less than 20 µm was observed in the gastrocnemius of WT and *mdx* mice in comparison to untreated mice, though no apparent differences between analyzed groups were revealed (Figure 6a). Nonetheless, when the total number of blood vessels in *mdx* mice was normalized to WT counterparts, a significant decline in their abundance was noticed on day 7 post-injury and remained diminished 21 days after FAL in comparison to untreated *mdx* animals (Figure 6b).

Importantly, a profound induction of CD105 (Figure 6c) and *Cxcr4* (Figure 6d), potent modulators of ischemia-induced angiogenesis/arteriogenesis [42,43], was noticed in WT, but not in *mdx* mice 7 days after FAL, whereas it returned to the basal level on day 21 after surgery. Concomitantly, though no upregulation of other crucial angiogenic factors, namely VEGF (Figure 6e) and stromal cell-derived factor-1 (SDF-1, Figure 6f), was observed both in WT and *mdx* mice as a result of HLI, VEGF was slightly increased in dystrophic mice on day 7 post-injury (Figure 6e), whereas SDF-1 additionally at day 21 after FAL (Figure 6f), when ischemic legs were compared.

### 3.4. HLI-Induced Inflammatory Response Is Impaired in 12-Week-Old Dystrophic Mice

**Figure 7:**
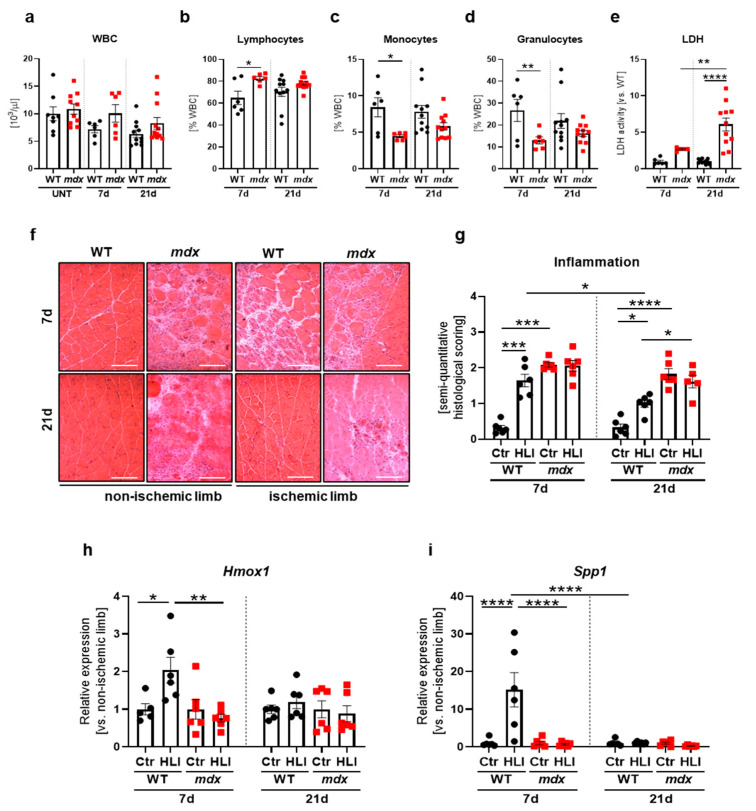
Inflammation markers following a hindlimb ischemia (HLI) procedure in 12-week-old dystrophic and WT animals. (**a**) The number of white blood cells (WBC) and (**b**) the percentage of lymphocytes, (**c**) granulocytes, and (**d**) monocytes assessed in the peripheral blood of WT and *mdx* mice, 7 and 21 days after HLI and in untreated (UNT) WT and *mdx* mice in case of WBC; blood cell count; *n* = 6–12/group. (**e**) Lactate dehydrogenase (LDH) release to serum analyzed 7 and 21 days after HLI in WT and *mdx* animals; activity assay; *n* = 6–12/group. (**f**) Representative pictures of hematoxylin and eosin staining of the gastrocnemius with (**g**) semi-quantitative histological scoring of inflammation in the non-ischemic and ischemic leg of WT and *mdx* mice, 7 and 21 days after HLI; microscopic assessment using a Nikon Eclipse microscope. Scale bar: 100 μm; *n* = 5–6/group. The expression of (**h**) *Hmox1* and (**i**) *Spp1* in the gastrocnemius of WT and *mdx* animals 7 and 21 days after HLI; qRT-PCR; *n* = 5–6/group. The results are presented as mean ± SEM. * *p* < 0.05, ** *p* < 0.01, *** *p* < 0.005, **** *p* < 0.0001 by one-way ANOVA with Tukey’s posthoc test.

HLI did not result in systemic inflammation as evidenced by normal white blood cells (WBC) count in the blood of WT and *mdx* mice both 7 and 21 days after ischemia induction in comparison to untreated animals of both genotypes (Figure 7a).

Nonetheless, when distinct subsets of immune cells were examined upon HLI conditions, a significant increase in the percentage of lymphocytes (Figure 7b) at the expense of monocytes (Figure 7c) and granulocytes (Figure 7d) was visible in *mdx* vs. WT animals 7 days after FAL, with no further changes at a later time point post-injury. Instead, the level of LDH activity was even more prominently increased in dystrophic mice 21 days after FAL in comparison to 7 days after surgery (Figure 7e).

We then assessed muscle inflammation based on hematoxylin and eosin (H&E)-stained sections of the gastrocnemius (Figure 7f). Markedly increased inflammatory cell infiltration was detected in the control, non-ischemic limbs of dystrophic vs. WT mice 7 and 21 days after HLI (Figure 7f, g). However, the induction of an inflammatory reaction as the result of FAL was noticed only in WT animals, predominantly 7 days after HLI, and it was reduced 21 days post-injury. *mdx* animals already maintained a high inflammatory status and failed to further respond to HLI induction, as no apparent differences between non-ischemic and ischemic limbs were noticed (Figure 7f,g). Similarly, substantial but transient upregulation of anti-inflammatory heme oxygenase-1 (*Hmox1*, Figure 7h), reported by us to exert a protective role in *mdx* animals [25], together with *Spp1* (Figure 7i), was demonstrated solely in WT mice.

### 3.5. 12-Week-Old Dystrophic Mice Failed to Upregulate a Regenerative and Tissue Remodeling Response to HLI

**Figure 8:**
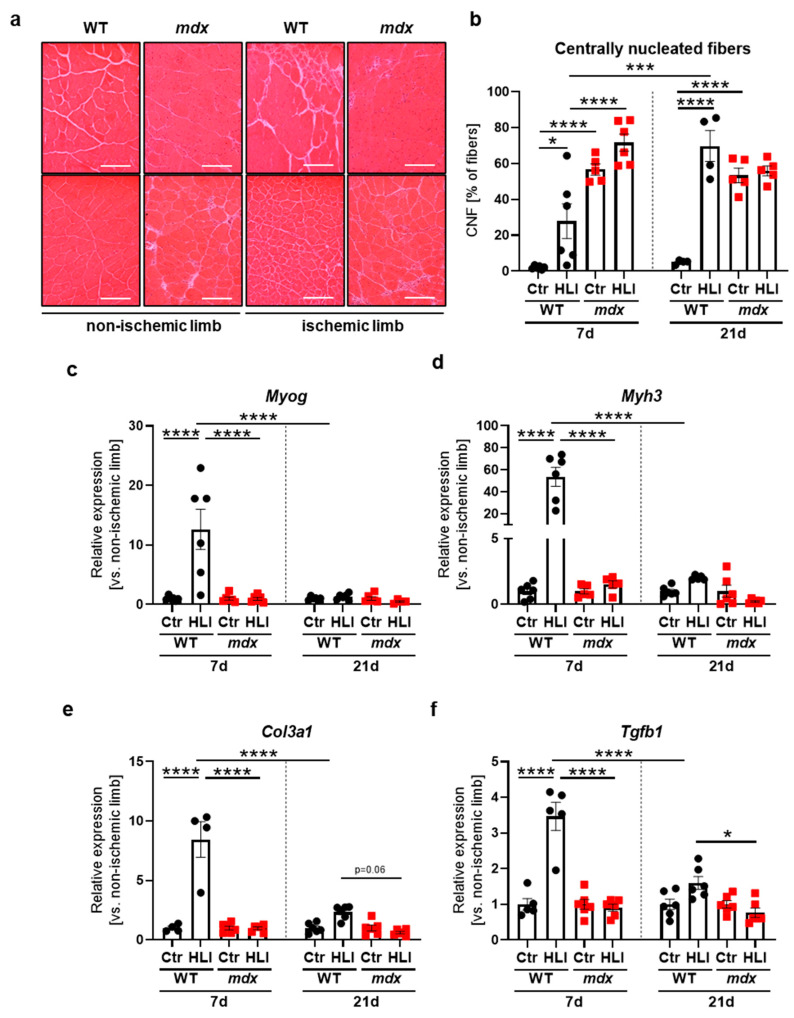
Regeneration and fibrotic markers following a hindlimb ischemia (HLI) procedure in 12-week-old WT and dystrophic animals. (**a**) Representative photos of H&E staining of centrally nucleated fibers in the non-ischemic (Ctr) and ischemic (HLI) tibialis anterior of WT and *mdx* mice 7 and 21 days after HLI together with (**b**) the calculated percentage of centrally nucleated fibers (CNF) among all fibers; representative pictures with a Nikon Eclipse microscope. Scale bar: 100 μm; *n* = 4–6/group. (**c**) The expression of regenerative markers, *Myog* and (**d**) *Myh3,* as well as fibrotic factors (**e**) *Col3a1* and (**f**) *Tgfb1* in the non-ischemic limb and the ischemic limb of the gastrocnemius muscle from WT and *mdx* mice 7 and 21 days after HLI. qRT-PCR; *n =* 4–6/group. The results are presented as mean ± SEM. * *p* < 0.05, *** *p* < 0.005, **** *p* < 0.0001 by one-way ANOVA with Tukey’s posthoc test.

To assess muscle fiber regeneration capacity after FAL, we counted centrally nucleated fibers (CNF) in the tibialis anterior based on H&E staining (Figure 8a). A detailed quantitative analysis of the whole muscle sections revealed an enormous upregulation of the number of CNF in control, a non-ischemic limb of *mdx* vs. WT mice both 7 and 21 days after FAL (Figure 8b), emphasizing constant damage/regeneration cycles in dystrophic muscles. Nonetheless, no further upregulation of the CNF percentage was detected in dystrophic animals when the ischemic limb was compared to non-ischemic at each analyzed time point after FAL (Figure 8b). On the contrary, WT animals triggered a prompt regenerative response as visualized by an elevated percentage of CNF already 7 days after HLI, which was even further intensified 21 days post-injury, reaching the level of non-ischemic and ischemic limbs of *mdx* mice (Figure 8b). Such a strong reaction in WT mice was also visible on mRNA level by the transient induction of pivotal players in myogenesis, namely *Myog* (Figure 8c) encoding myogenin which is expressed in regenerative skeletal muscles [44], as well as *Myh3* (Figure 8d) encoding embryonic myosin heavy chain isoform present in newly-formed myofibers [45]. In the case of *mdx* animals, however, both *Myog* and *Myh3* remained at a constant level, as no changes as a result of HLI were visible, despite their basal, profound upregulation in non-ischemic conditions in comparison to WT counterparts (Figure S3a,b).

The remodeling of the muscle tissue might also contribute to the proper regeneration and revascularization after HLI. Hence, we determined the expression of typical fibrotic genes, namely *Col3a1* (Figure 8e) and *Tgfb1* (Figure 8f), which exhibited a similar pattern—potent induction of both factors in response to HLI, was noticed in WT, but not in *mdx* mice 7 days after surgery, and was blunted to the basal level on day 21. *mdx* animals failed to further increase *Col3a1* (Figure 8e) and *Tgfb1* (Figure 8f), which at the basal level were both increased in the control, a non-ischemic limb of *mdx* vs. WT, 7 and 21 days after HLI (Figure S3c,d).

## 4. Discussion

Though dysregulation of angiogenesis was proposed to contribute to DMD pathology already in the pre-dystrophin era [11], and vascular-based approaches showed a promise in preclinical animal studies [21,46,47,48], no clear consensus about the angiogenesis status in *mdx* mice exists to date. A detailed description of the vascular changes associated with the age of *mdx* animals, along with the verification of published data, might help in planning studies that investigate the therapeutic potential of vascular therapies utilizing this most commonly used mouse model of DMD.

An evaluation of the angiogenic status in skeletal muscles of young, 6-week-old mice at the stage of robust degeneration/regeneration intervals [18,19] and older, 12-week-old mice, indicated that the most profound aberrations were present in the latter. This provides more evidence for the suggested influence of age on angiogenesis-related disturbances in DMD [11]. Though we initially assumed similar differences irrespectively of age, due to the comparable expression pattern of angiogenic factors related to VEGF signaling, CXCL12/SDF-1, and Ang1,2/Tie2 axes, it appeared that neither blood vessel number nor basal blood flow and functional recovery from HLI was altered in younger, 6-week-old dystrophic mice in comparison to age-matched WT mice. But all these parameters were affected in older, 12-week-old *mdx* vs. WT counterparts. It underlines the complexity of angiogenesis-related changes, which rather do not depend on the deficiency or excessive expression of a certain angiogenic stimulus, which was also proposed by Palladino et al. [15]. Accordingly, despite the decreased protein level of a major pro-angiogenic VEGF in skeletal muscles of 12-week-old *mdx* mice reported in our findings [22,23,24] and also those of others [47] previous reports, we observed an even higher number of CD31^+^α-SMA^+^ double-positive blood vessels in the gastrocnemius and diaphragm which were mostly located in the cell infiltration/muscle damage areas. Although this observation points toward inflammation-induced angiogenesis, it warrants further verification. Moreover, the influence of the cross-talk between SCs and ECs proposed recently to regulate vasculature in skeletal muscles cannot be ruled out and represents an interesting area for future work [49,50].

To the best of our knowledge, here, for the first time, we demonstrated the intensified vasculature in the diaphragm and gastrocnemius of 12-week-old dystrophic mice at the basal level with a slightly increased diameter of CD31^+^α-SMA^+^ double-positive blood vessels, possibly as the result of adaptation to changes in wall shear stress or tissue metabolic status. The observed effects regarding the abundance of CD31^+^α-SMA^+^ double-positive blood vessels might be muscle-specific, as no differences were found in the tibialis anterior. It has to be, however, emphasized that skeletal muscles are heterogeneous tissues, differing in the composition of slow-twitch and fast-twitch fibers, and fiber type-specific angiogenesis was demonstrated previously in response to endurance and might be related to mitochondrial quantity/content/biogenesis [51,52]. In contrast to our results, Matsakas et al. found reduced blood vessel abundance in the tibialis anterior of 6–8-week-old *mdx* mice, with normal blood flow in both the gastrocnemius and tibialis anterior (however, the background of *mdx* mice was not clearly indicated) [53]. On the other hand, Latroche et al. showed no profound abnormalities in microvessel organization with a decrease in terminal arteriole density in the gastrocnemius of 3-month-old *mdx*-4Cv mice accompanied with even enhanced muscle blood perfusion due to ischemic stress [16]. Those confounding results might be related to the age, *mdx* strains, genetic background, and, most importantly, methodological approaches, especially concerning the blood vessel markers used by different groups. It has to be pointed out that the histological analysis and quantification of dystrophic muscle vasculature is extremely challenging due to the robust infiltration of immune cells, which may also express some of the ECs markers. In our approach, we decided to double-stain blood vessels with CD31 and α-SMA. Hence, the quantification mostly covered and was limited to mature blood vessels within the muscles [54]. We did not focus on the quantification of small capillaries, nor did we examine structural differences of the large hindlimb vessels. Hence, the results from basal blood perfusion measurements of all the hindlimbs may not provide an explicit explanation to the alterations in the vasculature of the single muscle.

In line with the state-of-the-art histological assessment, a detailed flow cytometry analysis revealed an increased percentage of bona fide skeletal muscle ECs, defined as CD45^−^CD31^+^CD34^+^Sca-1^+^ cells (arterial, capillary, and venular ECs) [55] in dystrophic mice, together with a diminished percentage of pericytes, pivotal for the maintenance of blood vessels, as their deficiency may result in increased vascular permeability [56,57]. Taking into account published reports on defects in ECs in animal models of DMD and DMD patients related to both morphological alterations and properties [11], we put into the question proper functionality of *mdx* blood vessels, as reflected by diminished blood flow and blood flow recovery after FAL. Moreover, such an assumption might be strengthened by our unpublished data on human-induced pluripotent stem cells (hiPSC)-derived ECs displaying marked aberrations as the results of dystrophin loss. What is interesting is that studies on muscle biopsies from DMD patients indicated a greater area of capillaries and ECs even in preclinical DMD patients in comparison to controls and individuals suffering from other neuromuscular disorders [58]. Finally, Loufrani et al. demonstrated attenuated flow-induced vascular dilation related to mitigated mechanotransduction of shear stress, specifically at the surface of ECs in arteries of mice lacking dystrophin, emphasizing that this defect might result in deleterious consequences related to the angiogenesis process [59].

When we further subjected dystrophic animals to the HLI, we again noticed the age-dependent decline of the angiogenic response in dystrophic mice. In general, an impairment in angiogenesis as a function of age is known; nonetheless, in our study, WT animals at 6 and 12 weeks of age exhibited a comparable basal blood perfusion level as well as a restored blood flow to a similar degree after HLI, rather excluding the impact of age itself on the observed effects in dystrophic animals. Though Straino et al. demonstrated even enhanced hindlimb perfusion in 8-week-old *mdx* vs. WT mice 7 and 14 days after HLI, no differences were found at the end-point measurement 21 days after the surgery [14], similar to our results conducted on 6-week-old mice. An improved ischemic/non-ischemic limb perfusion ratio in *mdx* vs. WT counterparts with increased arterioles length density observed by Straino et al. was not accompanied by an elevation of capillaries/arterioles as a result of HLI itself in comparison to non-ischemic conditions in both genotypes [14]. The same group in another study by Palladino et al. reported a decreased blood flow in older 6-month-old dystrophic mice after HLI [15]. Despite methodological discrepancies (femoral artery dissection vs. FAL utilized in the present study), it might be concluded that in older 12-week-old *mdx* animals, the angiogenic response is compromised. On the other hand, in younger *mdx* mice, at the phase of the efficient regenerative response, blood flow recovery after femoral artery ligation/dissection is not perturbed.

When Straino et al. analyzed the angiogenic potential toward tubular structures formation in vitro by human umbilical vein endothelial cells, even an accelerated response was observed in the presence of serum from 8-week-old *mdx* mice [14]. On the contrary, in our similar experiment, the use of the serum from older 12-week-old mice led to impeded vessel-like structure organization by HAECs subjected to dystrophic mice-derived serum. This strongly suggests that the level of circulating angiogenic factors also vary between *mdx* and WT mice of different age. Moreover, we may suspect that either the higher level of anti-angiogenic factors and/or too low expression of pro-angiogenic ones in the serum of 12-week-old *mdx* mice contributes to the hampered restoration of blood flow after HLI in our experimental settings. Apart from that, it is worth mentioning that the recruitment of immune cells from the blood is an important determinant of the efficient recovery from HLI. The temporal increase in the level of circulating monocytes after femoral artery ligation is well described—they are recruited to the blood and subsequently to ischemic tissue in response to local production of certain cytokines/chemokines [60,61,62]. In our analysis, the monocytes percentage was decreased in *mdx* animals 7 days after HLI in comparison to WT counterparts, which could, at least partially, explain the impaired blood flow recovery after HLI. Concomitantly, a diminished level of granulocytes (neutrophils, eosinophils, and basophils) noticed in dystrophic mice possibly contributed to those effects, especially given the fact that neutrophils were demonstrated to play a pivotal role in tissue remodeling and angiogenesis in the ischemic limb [63]. Quite puzzlingly, at the same time, the level of lymphocytes, which also contribute significantly to vascular repair [64], was elevated in the blood of *mdx* mice 7 days after the HLI procedure. Although it is not fully clear to us and would require additional studies, we may presume that local production of certain cytokines/chemokines stimulating the recruitment of distinct immune cell population at the right time after HLI is altered upon the dystrophic condition, resulting in differences in the proportion of circulating lymphocytes and monocytes/granulocytes possibly affecting the recovery from HLI in dystrophic mice. Importantly, the activity of LDH in the serum, indicating the degree of tissue injury, was even higher in dystrophic mice 21 days vs. 7 days after HLI and may reflect the persistently high release of LDH not only from necrotic muscle fibers but also other cells, including immune cells infiltrating ischemic tissue.

Finally, we wanted to understand the skeletal muscle-related effects accompanying functional impairment in blood flow recovery of 12-week-old mice as a result of HLI. A similar and very clear pattern of changes might be appreciated when the analysis of markers related to angiogenesis, inflammation, tissue remodeling, and regeneration was performed. Namely, a blunted response to HLI at an early time-point after the surgery in dystrophic animals was apparent both when inflammation and CNF were scored based on histology, as well as when CD105, *Cxcr4*, *Hmox1*, *Spp1*, *Myh3*, *Myog*, *Tgfb1*, and *Col3a1* were analyzed either on the protein or mRNA level. These results, together with literature reports [65,66], suggest that prompt temporal activation of molecular events orchestrates proper restoration of blood flow. Dystrophic animals fail to respond to HLI, as in all of the performed analyses, no differences between ischemic and non-ischemic limbs were visible. At the same time, it has to be stressed that the majority of analyzed factors are already upregulated in *mdx* animals in a steady state, as we have shown in the present and previous works [22,23,24,25]. As an example, here, we demonstrated that *Spp1* is enormously elevated in the gastrocnemius of untreated dystrophic mice. Though *Spp1* was reported to aggravate the dystrophic phenotype [37], its essential role in recovery from hindlimb ischemia has also been emphasized [67]. As such, a very prominent but transient increase was noticed only in WT animals 7 days after HLI, indicating that temporal, physiological augmentation of *Spp1* is repressed by the already exacerbated pathological, dystrophic phenotype. Even more strikingly, we indicated strong upregulation of *Myh3* in *mdx* mice (~50 times vs. WT) in basal, non-HLI conditions. This was comparable to the induced *Myh3* in ischemic vs. non-ischemic limb of WT animals 7 days after HLI. Despite that, functional recovery from HLI was noticed only in non-dystrophic mice.

At the same time, we undermined the importance of VEGF and SDF-1 in HLI-induced neovascularization, at least in our experimental settings, as no increase in the protein level of those factors at any time-point after HLI was noticed in both WT and *mdx* animals when ischemic vs. non-ischemic limbs were compared. In contrast, Palladino et al. demonstrated quite profound upregulation of VEGF 7 days after femoral artery dissection with no differences between WT and *mdx* animals [15]. Nonetheless, many contradictory reports on the role of VEGF in limb ischemia and vessel regeneration influenced by numerous factors and conditions have already been described [68]. Instead, we pointed toward the roles of the SDF-1 receptor, *Cxcr4,* and endoglin (CD105) in the proper restoration of blood flow after HLI, suggested already by others [42,43].

In light of all the above, the silent finding of this study indicates that dystrophic animals at 12 weeks of age fail to physiologically recover from HLI, suggesting that chronic and severe DMD pathology is already pushed to the limit and may not properly respond to the additional challenge, as shown by us in vascular-based settings.

## 5. Conclusions

In conclusion, we provided another piece of evidence indicating an age-dependent effect on angiogenesis in dystrophic animals. As impeded blood flow restoration was revealed in 12-week-old but not 6-week-old dystrophic mice, we propose that older *mdx* mice should be used in preclinical studies to evaluate the impact of vascular-based therapies on the restoration of functional angiogenesis to mitigate DMD severity.

## Figures and Tables

**Table 1:** The sequences of forward (F) and reverse (R) primers used in quantitative real-time PCR (qRT-PCR.)

Gene	Full Gene Name	Sequence 5′-3′
	Mouse primers	
*Ang 1*	Angiopoietin 1	F:CAGTGGCTGCAAAAACTTGA R:TGGGCCATCTCCGACTTCAT
*Ang2*	Angiopoietin 2	F:CTCTTCTTTACGGATAGCAA R:AGCCACGGTCAACAACTCGC
*Col3a1*	Collagen type III alpha 1 chain	F:ATCTATGAATGGTGGTTTTCA R:TTTTGCAGTGGTATGTAATGT
*Cxcl1*	C-X-C motif chemokine ligand 1	F:AAAGATGCTAAAAGGTGTCC R:GTATAGTGTTGTCAGAAGCC
*Cxcl12*	C-X-C motif chemokine ligand 12	F:CCTTCAGATTGTTGCACGGCT R:CCCACCACTGCCCTTGCATC
*Cxcr4*	C-X-C chemokine receptor type 4	F:AAACCTCTGAGGCGTTTGGT R:AGCAGGGTTCCTTGTTGGAG
*Eef2*	Eukaryotic elongation factor 2	F:AGAACATATTATTGCTGGCG R:AACAGGGTCAGATTTCTTG
*Hmox1*	Heme oxygenase 1	F:CCTCACTGGCAGGAAATCATC R:CCTCGTGGAGACGCTTTACATA
*Kdr*	Kinase insert domain receptor	F:CGGCCAAGTGATTGAGGCAG R:ATGAGGGCTCGATGCTCGCT
*Mmp11*	Matrix metalloproteinase 11	F:CAGATTTGGTTCTTCCAAGG R:AGATCTTGTTCTTCTCAGGAC
*Myog*	Myogenin	F:CAGTACATTGAGCGCCTACAG R:GGACCGAACTCCAGTGCAT
*Myh3*	Myosin heavy chain 3	F:TCTAGCCGGATGGTGGTCC R:GAATTGTCAGGAGCCACGAA
*Spp1*	Secreted phosphoprotein 1	F:CCATCTCAGAAGCAGAATCTCCTT R:GGTCATGGCTTTCATTGGAATT
*Tgfb1*	Transforming growth factor beta 1	F:GGATACCAACTATTGCTTGAG R:TGTCCAGGCTCCAAATATAG
*Tie2*	Tek receptor tyrosine kinase	F:TGTCCAAAGGAGAATGGCTC R:GGCGGCATCCATCCGTAACC
	Human primers	
*EEF2*	Eukaryotic elongation factor 2	F:GAGATCCAGTGTCCAGAGCAG R:CTCGTTGACGGGCAGATAGG
*VEGF*	Vascular endothelial growth factor A	F:AAGGAGGAGGGCAGAATCAT R:CTCAGTGGGCACACACTCCA

## Data Availability

Not applicable.

## Supplementary Materials

The following are available online at https://www.mdpi.com/article/10.3390/biomedicines9050481/s1, Supplementary Figure S1: General characteristics of 12-week-old dystrophic animals; Supplementary Figure S2: Gating strategy for flow cytometry analysis of fibro-adipogenic progenitors (FAPs), endothelial cells (ECs), and pericytes; Supplementary Figure S3: The mRNA level of genes associated with regeneration and fibrosis in 12-week-old WT and dystrophic animals.
