# Supplementary material for: Age-Dependent Dysregulation of Muscle Vasculature and Blood Flow Recovery after Hindlimb Ischemia in the mdx Model of Duchenne Muscular Dystrophy
Source: Biomedicines. 2021 Apr 27;9(5):481. doi: 10.3390/biomedicines9050481 (PMC8145677; doi:10.3390/biomedicines9050481)
Supplement: Supplementary file 1 [file biomedicines-09-00481-s001.zip › biomedicines-1189201-supplementary.pdf]

biomedicines

MDPI

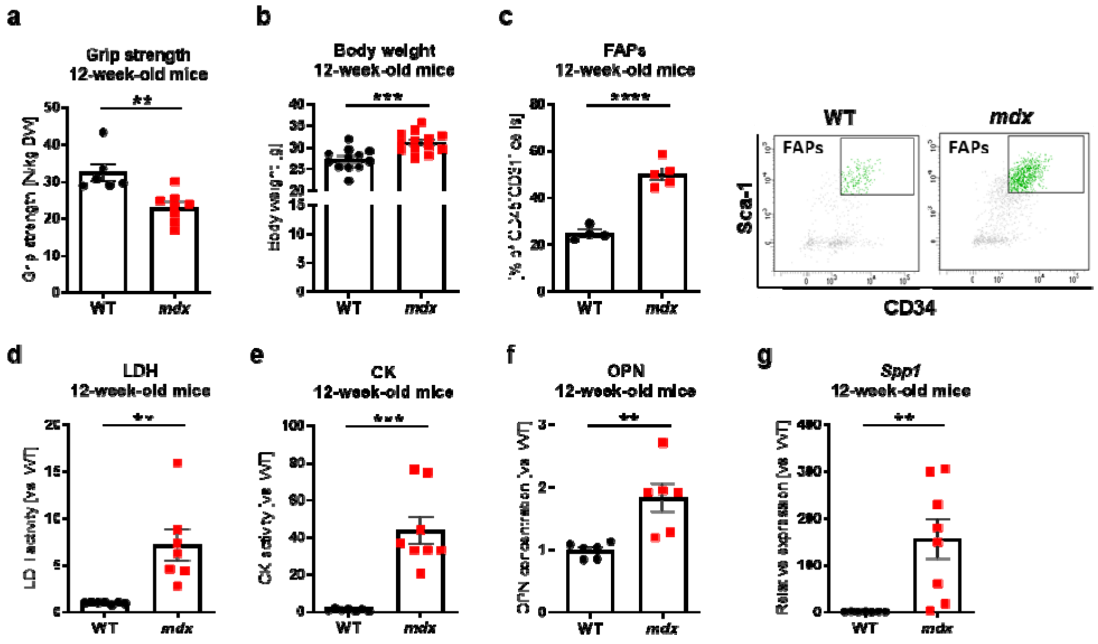

Figure S1. General characteristics of 12-week-old dystrophic animals. (**a**) *Mdx* mice demonstrate decreased muscle strength; forelimb grip strength test, $n = 6–8$/group; and (**b**) increased body weight; $n = 12–13$/group. (**c**) An abundance of fibro-adipogenic progenitors (FAPs) identified as $CD45^{-}CD31^{-}Sca\text{-}1^{+}CD34^{+}$ cells was higher in dystrophic hindlimb muscles; flow cytometry analysis calculated as a percentage of $CD45^{-}CD31^{-}$ cells and representative two-parameters flow cytometry dot plots; $n = 4–5$/group. (**d**) The level of markers of muscle damage in serum, lactate dehydrogenase (LDH)**,** and (**e**) creatine kinase (CK) is increased in *mdx* mice; activity assay; $n = 7–8$/group. (**f**) An elevated protein level of osteopontin (OPN) in serum ELISA; $n = 6$/group and (**g**) its mRNA transcript (*Spp1*) in the gastrocnemius of dystrophic mice; qRT-PCR; $n = 7–8$/group. The results are presented as mean ± SEM. ** $p < 0.01$, *** $p < 0.005$, **** $p < 0.0001$ with unpaired two-tailed Student's t-test.

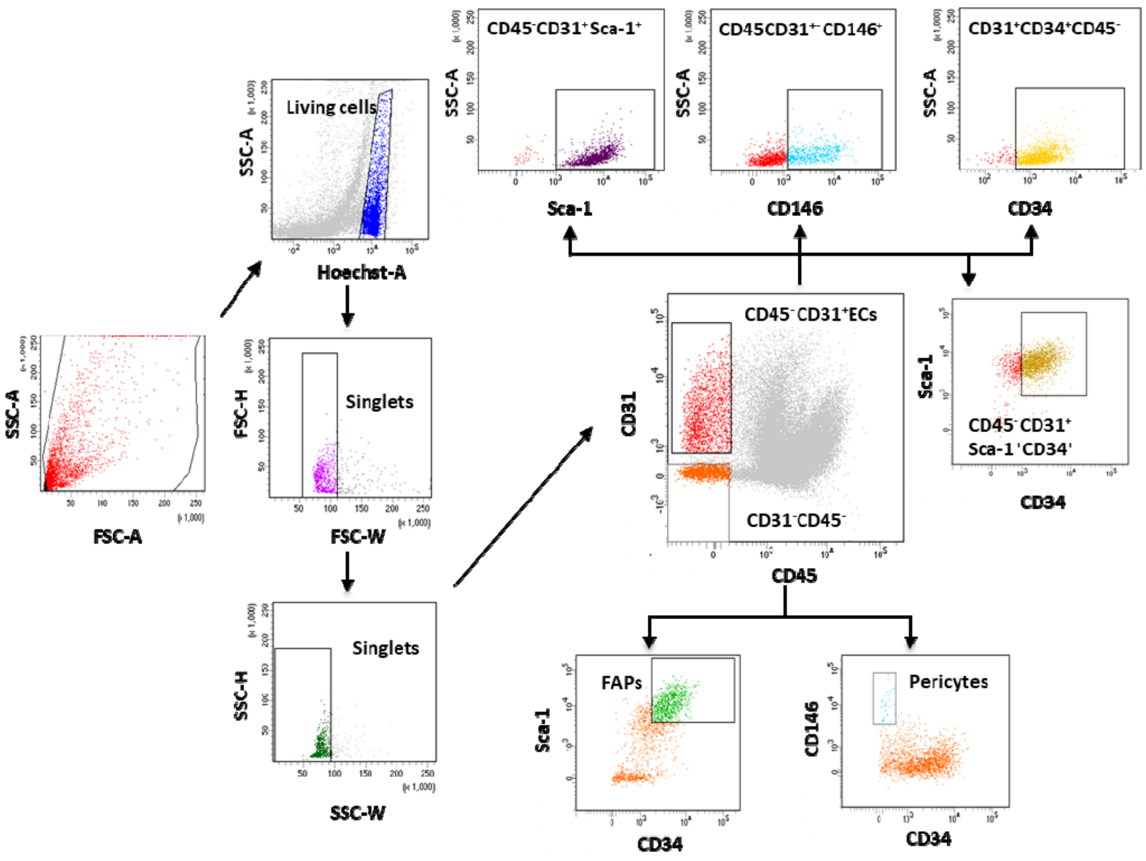

Figure S2. Gating strategy for a flow cytometry analysis of fibro-adipogenic progenitors (FAPs), endothelial cells (ECs), and pericytes.

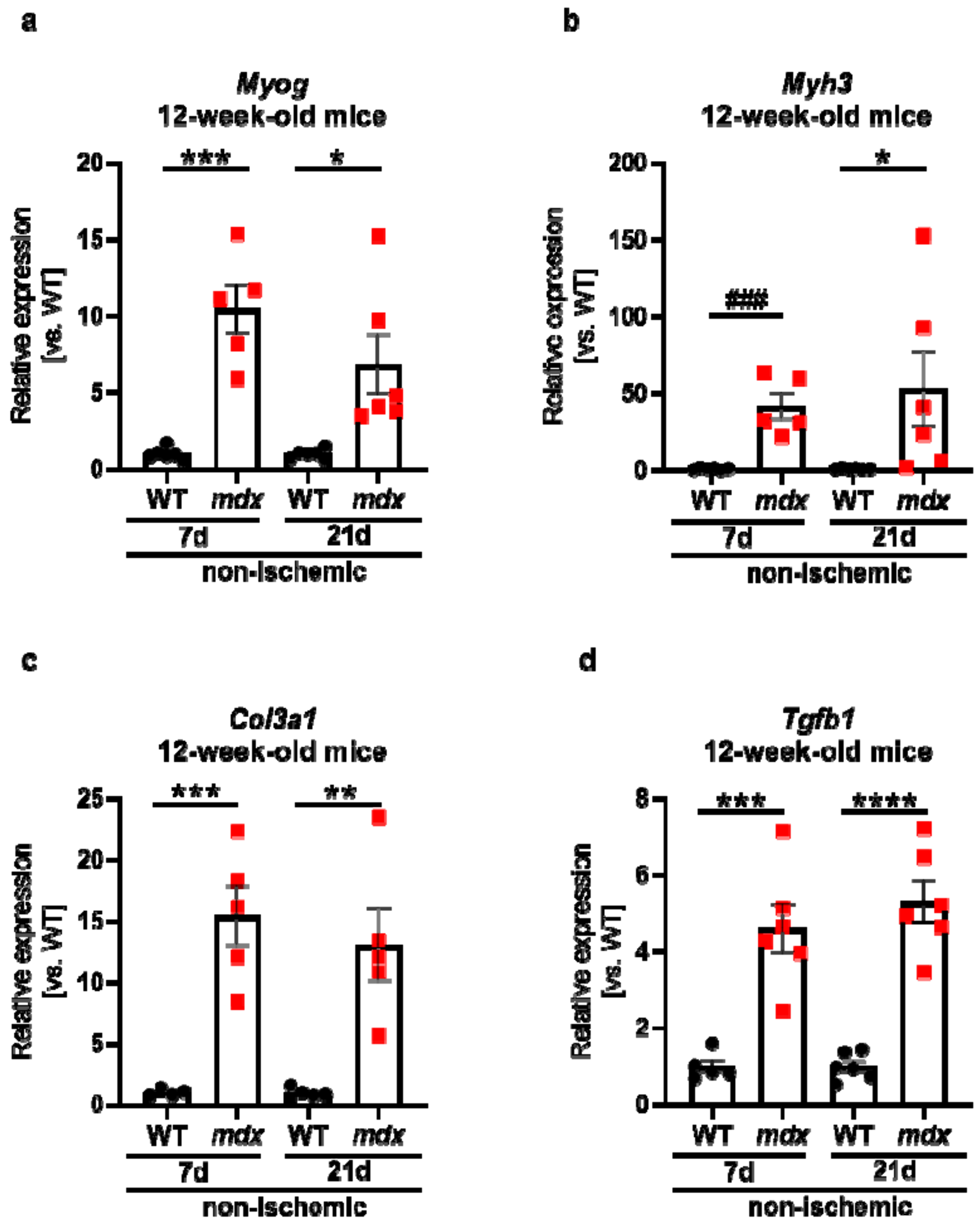

Figure S3.–The mRNA level of genes associated with regeneration and fibrosis in 12-week-old WT and dystrophic animals. The expression of regenerative markers, (**a**) *Myog* and (**b**) *Myh3* as well as fibrotic factors (**c**) *Col3a1* and (**d**) *Tgfb1* in the non-ischemic limb of WT and mdx mice 7 and 21 days after HLI. qRT-PCR; n = 4–6/group. The results are presented as mean ± SEM. * $p < 0.05$, ** $p < 0.01$, *** $p < 0.005$, **** $p < 0.0001$ by one-way ANOVA with Tukey's post-hoc test and ### $p <$ 0.005 with unpaired two-tailed Student's t-test.
